# Giant right ventricular thrombus as the revealing form of Behçet's disease

**DOI:** 10.1186/s43044-023-00354-5

**Published:** 2023-04-11

**Authors:** Anass A. Maaroufi, Sara S. Abouradi, Salah S. Hayar, Abdenacer A. Drighil

**Affiliations:** Cardiology Department, Ibn Rochd University Hospital, 20250 Casablanca, Morocco

**Keywords:** Behçet disease, Intra cardiac thrombus, Cardio-Behçet, Pulmonary embolism

## Abstract

**Background:**

Behçet's disease BD is a rare multisystemic disease, with rare cardiac involvement. This case illustrates a rare cardiac involvement as a giant intracavitary thrombus which was the revealing form of Behçet disease.

**Case presentation:**

An 15-year-old male admitted to the emergency department for progressive dyspnoea, hemoptysis for which an echocardiogram displayed a large echogenic mass in the right ventricle and angio CT revealed associated bilateral pulmonary embolism. The patient was then proposed for surgery for removal and pathological study the later confirmed its fibrin thrombotic nature. Behçet disease was suspected based on past history of recurrent oral aphthosis and confirmed with a positive pathergy test. Further management by anticoagulants, immunosuppressants and corticosteroids seemed effective to avoid relapse.

**Conclusions:**

Cardiac involvement during BD can be life-threatening as it is not always diagnosed in timely manner. However, intracardiac thrombus is uncommon with only few case reports. Echocardiography is the key tool for the diagnosis of intracardiac thrombus.

## Background

Behçet's disease BD is a rare chronic multisystemic disease with various systems involvement as: skin, joints, nervous, respiratory, gastrointestinal, and cardiovascular systems [1].

BD has a particular distribution worldwide, particularly in Japan and Mediterranean areas [2]. Although the vascular lesions are frequently observed in this disease, the cardiac involvement is rare and associated with the poor prognosis [1, 3].

We report a rare case of an adolescent, who presented initially a right ventricle thrombus that was the revealing form of Behçet’s Disease.

## Case presentation

We report the case of a 15-year-old male, admitted to the emergency department for hemoptysis of less than 100 ml of blood and worsening of dyspnea evolving from 1 month along with dry cough. The patient displayed breathlessness.

On physical examination, blood pressure was 115/75 mm Hg, respiration rate was 31 per minute, and O2 saturation at ambient air was 92%, with fever of 38 °C. There was no murmur on cardiac examination, with no clinical signs of deep venous thrombosis on calf examination and ECG showed sinus tachycardia of 110 bpm.

The biological parameters revealed mild microcytic anemia with hemoglobin concentration of 10 g/dl, neutrophil leukocytosis (12,000/mL). Liver and kidney function tests were normal. C reactive protein concentration was elevated at 122 mg/l.

Chest X-rays showed bilateral opacities with basal consolidation.

**Fig. 1:**
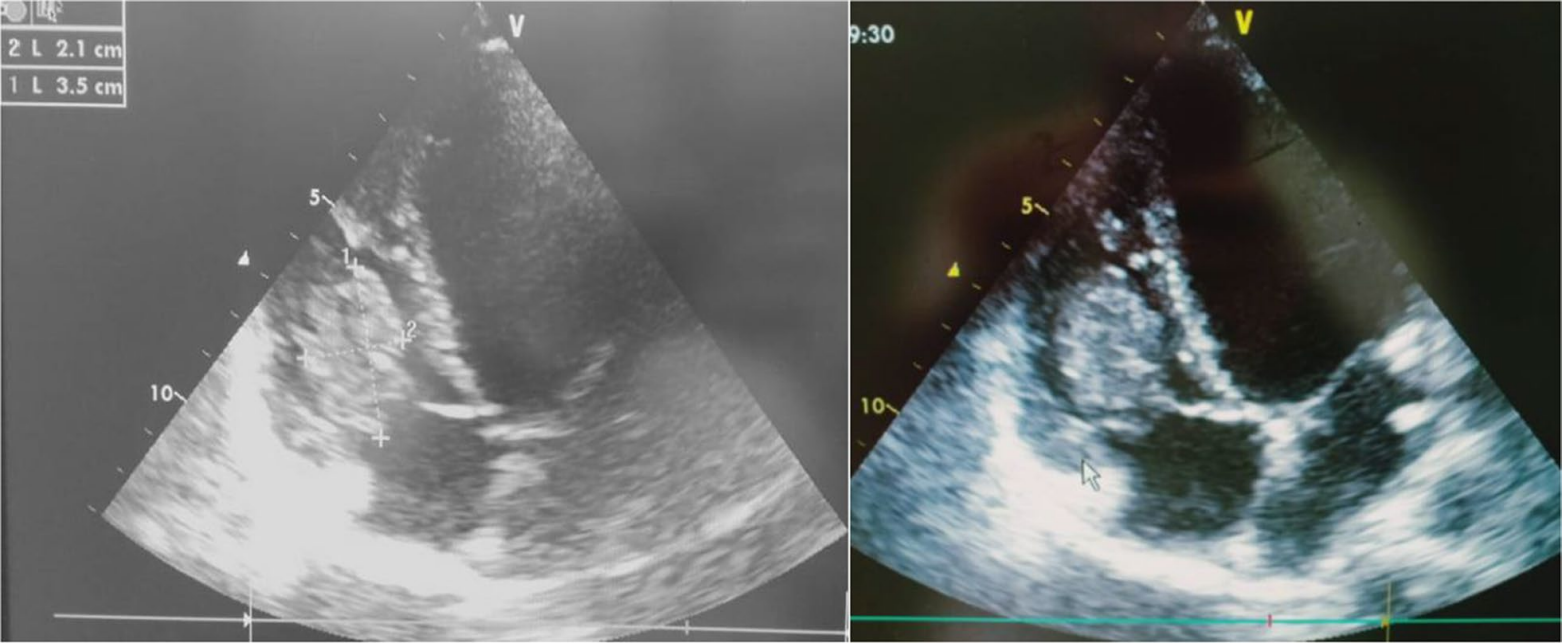
Transthoracic echocardiography: 4-chamber view showing a right ventricle mass attached to the anterior cusp of the tricuspid valve

We performed a transthoracic echocardiography, revealing a non-dilated left ventricle with preserved systolic function, a right ventricle moderately dilated of preserved function; with the presence of an homogeneous, echogenic mass of 31 × 21 mm with limited mobility attached to the base of the anterior tricuspid ring (Fig. 1).

**Fig. 2:**
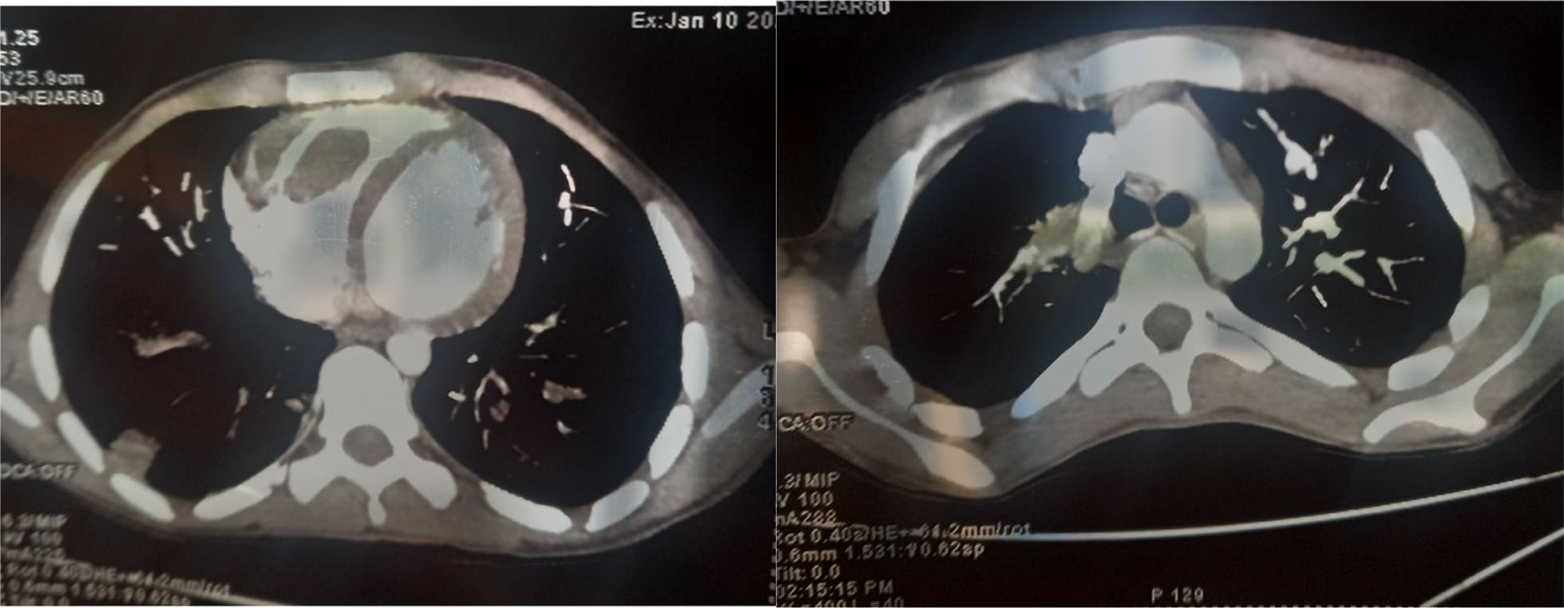
Thoracic Angio CT: bilateral pulmonary embolism associated with right ventricular mass

Furthermore, a angio thoracic-CT was ordered, which confirmed the diagnosis of bilateral pulmonary embolism along with bilateral peripheral wedge shaped opacities, suspecting associated pulmonary infarction moreover, it displayed the presence of a tissular-density formation in right ventricular cavity (Fig. 2).

At this stage, we suspected the diagnosis of intracardiac thrombus, still the ventricular localization of the mass is unusual, in the absence of known thromboembolic risk factors and the young age of patient. We proposed the patient for surgical mass removal, which was performed successfully with complete resection of a tissular mass: the macroscopic evaluation displayed a white pediculated mass associated with a micro-thrombi on its surface.

**Fig. 3:**
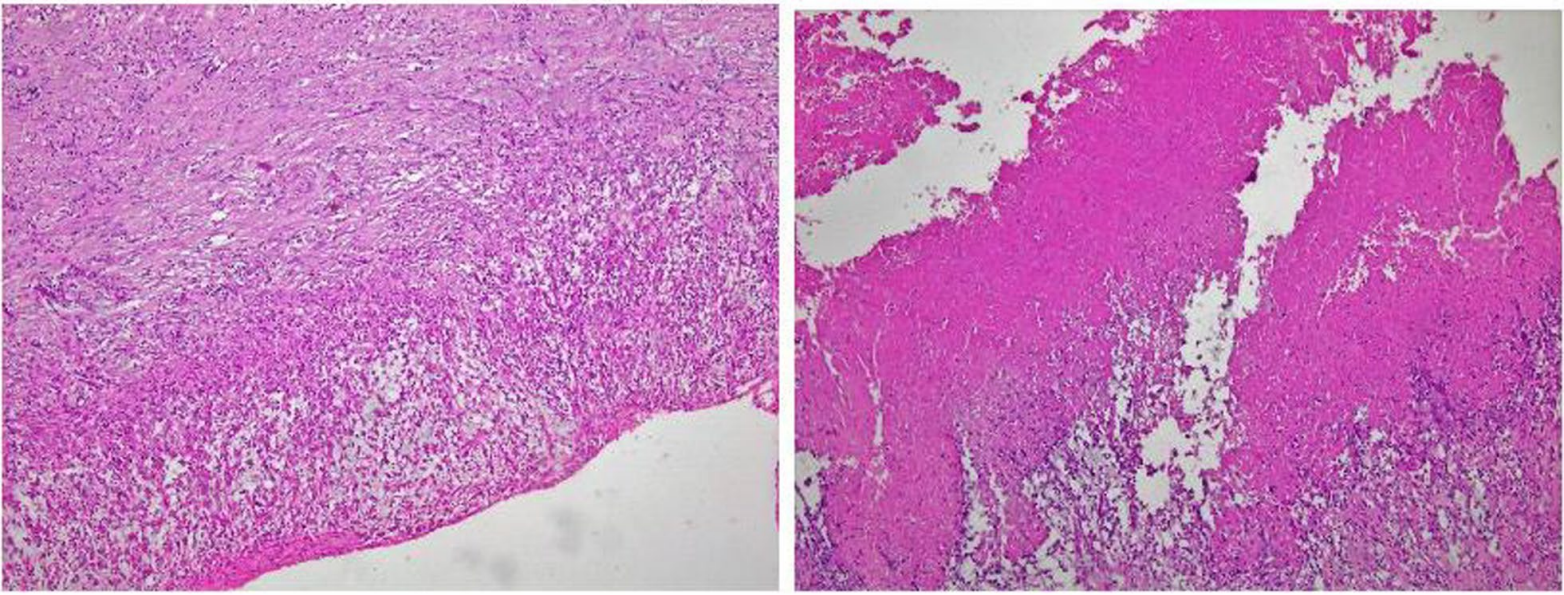
Histopathological images with fibrin rich leukocytic material adherent to cardiac wall suggesting intracardiac thrombus (standard coloration H&E on light microscope)

The histopathological examination confirmed the diagnosis of intracardiac thrombus by revealing a fibrin rich leukocytic material adhering to muscle tissue compatible with an adherent thrombus (Fig. 3).

In the course of the etiological investigation, the notion of recurrent oral aphthosis was discovered retrospectively. Then, a pathergy test was performed, which came back positive; hence, the diagnosis of Behçet's disease was confirmed.

With conjunction with internists, we treated the patient with a triple therapy based on azathioprine, prednisone and anticoagulation by anti-vitamin K. The clinical outcome was good, without any remarkable relapse or recurrence on further follow-up during 2 years.

Behçet’s disease is an inflammatory multisystemic disease with a particular geographic distribution, due mainly to the prevalence of HLA B 5 and HLA B 51 [4].

Often challenging to diagnose, without a definitive diagnostic laboratory test, diagnosis is based on characteristic clinical features:

- Orogenital ulceration
- Uveitis
- Joint or skin lesions
- CNS, or cardiac involvement.

Cardiac involvement during BD can be life-threatening as it’s not always diagnosed in timely manner or treated properly. It is often associated with reactive acute pericarditis related to inflammatory flare, and acute myocarditis has also been reported in literature.

However, Intracardiac thrombus formation with or without pulmonary emboli, a rare phenomenon, was reported in few previous case reports [5].

There are different hypotheses about pathophysiology of thrombus formation in Behçet’s disease as: increased blood viscosity related to endothelial dysfunction and systemic inflammation.

Endothelium damage due to pro inflammatory blood components and vasculitis of vasa vasorum are related to degeneracy of elastic vascular structures and could result in aneurysms and slows blood flow [6].

Increased procoagulants factors as VIII and circulating von Willebrand factor were noted in BD patients [7].

The clinical signs of intracardiac thrombus remain non-specific with dyspnea, hemoptysis or simple fever.

It is often associated with concomitant thrombosis of the vena cava or complicated with pulmonary embolism (41%), or pulmonary artery aneurysms (38%) [8], which is often related to a relative high mortality rate [9].

The thrombus is most often located in the right heart. The right ventricle remains the most frequent location, followed by the right atrium. There were only a few cases in which the involvement of left chambers has been reported.

Echocardiography is the key tool for the diagnosis of intracardiac thrombus, as it usually shows the presence of a hyperechoic, homogeneous, well-limited, immobile formation attached to the atrium or ventricle with a broad base [10].

The echocardiographic appearances of large intracardiac thrombosis can be misdiagnosed as a primary heart tumor or a large vegetation in the context of infectious endocarditis. In difficult cases, histological examination of a surgical specimen can be used to decide [10]. Our patient had no history of BD, no signs of infective endocarditis. A surgical resection of the mass was proposed to eliminate the diagnosis of a primary tumor.

Other imaging tests as CT and MRI could be helpful in the diagnosis and the assessment of associated thoracic manifestations of Behçet’s Disease as systemic thrombosis and Pulmonary artery aneurysm (PAA).

Therapeutic management of BD’s vascular involvement is not well codified. Indeed, it must take into account the severity of the vascular damage. Immunosuppressive drugs associated with corticosteroids are the cornerstone of the treatment. Anticoagulation, on the other hand, remains to be debated [11]. Several studies have shown the inefficacy of anticoagulant alone in preventing venous thrombosis. However, the triple association of anticoagulants, immunosuppressants and corticosteroids seems to be the most effective choice in intracardiac thrombosis as confirmed in several retrospective studies [12].

## Conclusions

This case report provides an example of a rare but serious Behçet’s disease manifestation, related to a massive right ventricular thrombus as the revealing form of Behçet’s disease and complicated by the bilateral pulmonary embolism.

The displayed findings advocate the search for features compatible with Behçet’s disease in case of right heart masses. On the other hand, an early transthoracic echocardiography seems reasonable in patients with Behçet’s disease in order to diagnose any cardiovascular involvement.

## Data Availability

All data and materials related to this report are accessible at any time upon request.

## Abbreviations

BD: Behçet’s disease
CNS: Central nervous system

## References

[1] Alli N, Gur G, Yalcin B, Hayran M (2009). Patient characteristics in Behçet disease. Am J Clin Dermatol.

[2] Leonardo NM, McNeil J (2015). Behçet’s disease: is there geographical variation? A review far from the Silk Road. Int J Rheumatol.

[3] Marzban M, Mandegar MH, Karimi A, Abbasi K, Movahedi N, Navabi MA (2008). Cardiac and great vessel involvement in “Behçet’s disease”. J Card Surg.

[4] Mizuki N, Inoko H, Ohno S (1997). Pathogenic gene responsible for the predisposition to Behçet’s disease. Int Rev Immunol.

[5] Özkan M, Emel O, Özdemir M, Yurdakul S, Kocak H, Özdogan H (1992). M-mode, 2-D and Doppler echocardiographic study in 65 patients with Behçet’s syndrome. Eur Heart J.

[6] Lippi G, Franchini M (2008) Pathogenesis of venous thromboembolism: when the cup runneth over. In: Seminars in thrombosis and hemostasis. Thieme Medical Publishers, pp 747–761 10.1055/s-0029-1145257 19214913

[7] Özoran K, Düzgun N, Gürler A (1995). Plasma von Willebrand factor, tissue plasminogen activator, plasminogen activator inhibitor, and antithrombin III levels in Behçet's disease. Scand J Rheumatol.

[8] Amchich Y, Reguig N, Boucaid A, Belghoule R, Zegmout A, Bourkadi JE (2020). Intracardiac thrombus in Behçet’s disease: a rare case in Morocco. Pan Afr Med J.

[9] Darie C, Knezinsky M, Demolombe-Rague S, Pinede L, Perinetti M, Ninet JF (2005). Cardiac pseudotumor revealing Behçet’s disease. Rev Med Intern.

[10] Ben Ghorbel I, Ibn Elhadj Z, Khanfir M, Braham A, Fekih M, Drissa H (2004). Thrombus intracardiaque au cours de la maladie de Behçet. J Mal Vasc.

[11] Schmitz-Huebner U, Knop J (1984). Evidence for an endothelial cell dysfunction in association with Behçet’s disease. Thromb Res.

[12] Alpsoy E, Leccese P, Emmi G, Ohno S (2021). Treatment of Behçet’s disease: an algorithmic multidisciplinary approach. Front Med.

